# *synapse*: interactive support on photoemission spectroscopy measurement and analysis for non-expert users

**DOI:** 10.1107/S1600577523008305

**Published:** 2023-10-27

**Authors:** Takuma Masuda, Masaki Kobayashi, Koji Yatani

**Affiliations:** a Department of Electrical Engineering and Information Systems, The University of Tokyo, 7-3-1 Hongo, Bunkyo-ku, Tokyo 113-8656, Japan; b Center for Spintronics Research Network, The University of Tokyo, 7-3-1 Hongo, Bunkyo-ku, Tokyo 113-8656, Japan; Uppsala University, Sweden

**Keywords:** photoemission spectroscopy, synchrotron radiation, user interface, data analysis

## Abstract

Photoemission spectroscopy, an experimental method based on the photoelectric effect, is now an indispensable technique used in various fields such as materials science, life science, medicine and nanotechnology. However, part of the experimental process of photoemission spectroscopy relies on experience and intuition, which is obviously a problem for novice users. In particular, photoemission spectroscopy experiments using high-brilliance synchrotron radiation as a light source are not easy for novice users because measurements must be performed quickly and accurately as scheduled within a limited experimental period. In addition, research on the application of information science methods to quantum data measurement, such as photoemission spectroscopy, is mainly aimed at the development of analysis methods, and few attempts have been made to clarify the problems faced by users who lack experience. In this study, the problems faced by novice users of photoemission spectroscopy are identified, and a native application named *synapse* with functions to solve these problems is implemented and evaluated qualitatively and quantitatively. This paper describes the contents of an interview survey, the functional design and the implementation of the application *synapse* based on the interview survey, and results and discussion of the evaluation experiment.

## Abstract 2

Problems faced by novice users of photoemission spectroscopy are identified, and a native application named *synapse* with functions to solve these problems is implemented and evaluated qualitatively and quantitatively.

## 1. Introduction

Photoemission spectroscopy is now indispensable in various fields such as materials science, life science, medicine and nanotechnology (Reinert & Hüfner, 2005 ▸; Winter & Faubel, 2006 ▸; Peles & Simon, 2009 ▸; Vilmercati *et al.*, 2009 ▸; Kobayashi, 2009 ▸; Papp & Steinrück, 2013 ▸; Lv *et al.*, 2019 ▸). However, part of the experimental process of photoemission spectroscopy relies on experience and intuition, making it difficult for novice users to understand. For example, while photoemission spectroscopy experimenters repeat measurements under the same conditions and aggregate the results to increase the signal-to-noise ratio (SNR) of a spectrum, they decide on how many iterations they will need both visually and empirically. Given the increasing number of users of photoemission spectroscopy across the boundaries between academia and industry, photoemission spectroscopy should be easy to understand even for non-skilled users.

When X-rays of energy higher than the work function are incident on a material, electrons are emitted from the surface via the photoelectric effect. The emitted electrons are called photoelectrons. Photoemission spectroscopy is used to analyze the kinetic energy and momentum distribution of these photoelectrons. Using photoemission spectroscopy, one can obtain information on the electronic states of materials.

In photoemission spectroscopy, synchrotron radiation is frequently used as a light source. The use of synchrotron radiation as a light source increases the SNR in a short measuring time due to its highly brilliant X-rays and improves measurement accuracy dramatically. In addition, for saving the beam time, users require fewer iterations for the measurement, and the measurement time is expected to be reduced. Since the photon energy of synchrotron radiation is tunable, one can measure spectra with varying photon energy. In particular, resonant photoemission spectroscopy near an absorption edge enables us to extract the partial density of states derived from a specific orbital related to the core-hole excitation (Fano, 1961 ▸). For these reasons, synchrotron radiation light sources are often used for photoemission spectroscopy.

However, while synchrotron radiation light sources are becoming increasingly popular, photoemission spectroscopy experiments using synchrotron radiation are particularly burdensome for unfamiliar users. Public use of these facilities is generally limited to a few times a year for a few hours to a few days each time, during which experimental teams must repeat scheduled measurements. In other words, users need to perform their scheduled experiments without failure in the limited time available. In addition, users must analyze spectra and consider the next measurement in a relatively short measurement time, which requires more accurate and quicker processing than in a laboratory experiment. This is especially difficult for users who are unfamiliar with the facility, equipment and experimental techniques.

In this study, we propose *synapse* (*synchrotron radiation app for photoemission spectroscopy experiment*), a measurement and analysis support system for novice users of photoemission spectroscopy. To build the system, we first conducted interviews with students and experts to clarify certain issues. Next, we formulated three features that should be implemented. Finally, we built an application with those functions and evaluated it quantitatively and qualitatively. In this paper, related works will be described first, followed by functions, implementation, evaluations, discussion and conclusion.

## 2. Related works

### 2.1. Use of informatics in quantum beam measurement

Various measurements have been made using beams of photons, neutrons and other quanta. In recent years, informatics methods have become prevalent in such quantum beam measurements including photoemission spectroscopy, motivated by the need to reduce measurement time (Ueno *et al.*, 2018 ▸, 2021 ▸) and to help users calculate (Devereaux *et al.*, 2021 ▸) or to carry out simulations (Rakitin *et al.*, 2018 ▸; Suzuki *et al.*, 2019 ▸). For example, Ueno *et al.* (2018 ▸) proposed an adaptive experimental design method and developed a machine-learning method for fitting curves to spectral plots, reducing the number of plots required for measurement. They also determined the convergence of iterative measurements necessary to increase the SNR and developed a specific method to reduce the number of unnecessary iterations (Ueno *et al.*, 2021 ▸). In addition, there are further examples of quantum beam measurements such as X-ray absorption spectroscopy (Suzuki *et al.*, 2019 ▸; Timoshenko & Frenkel, 2019 ▸) and X-ray diffraction (Hou *et al.*, 2019 ▸), some of which leverage machine learning. These attempts are called measurement informatics (Ueno *et al.*, 2018 ▸) or materials informatics (Rajan, 2005 ▸), which is one of the areas receiving a lot of attention lately. It has been pointed out that, for the application of informatics methods to materials science, the design of the platform is as important as the construction of the database and the application of machine learning (Takahashi & Tanaka, 2016 ▸). However, few studies have identified the problems that novices struggle with before design. Therefore, in this study, we first interviewed students and experts who were in a position to educate them to identify what novice users of photoelectron spectroscopy have trouble with.

### 2.2. Support by user interface

There have been many attempts to assist users by building interfaces. For example, *WIFIP* (Sallaz-Damaz & Ferrer, 2017 ▸) provides a remote-control interface for evolving devices; *Xi-cam* (Pandolfi *et al.*, 2018 ▸) enables users to organize, view and analyze image data with plugins. However, as mentioned earlier, there are few examples that identify and solve the problems that users of photoemission spectroscopy face in measurement and analysis. In particular, many existing interfaces focus on simply displaying spectra, processing and analyzing data, or connecting hardware and software.

In addition, Web-based user interfaces, such as *Web-Ice* (González *et al.*, 2008 ▸), *WIFIP* (Sallaz-Damaz & Ferrer, 2017 ▸), *Sirepo* (Rakitin *et al.*, 2018 ▸) and *Daiquiri* (Fisher *et al.*, 2021 ▸) are becoming more and more common these days. This is presumably because using a technology stack like that used for the Web saves time and effort in building applications. On the other hand, applications that run in a Web browser may use a command-line user interface to start up, making them difficult for some users to use. Therefore, in this study, we decided to use *Electron*, a framework that can build native applications without operating system dependence, to benefit from a Web-based technology stack and complete everything on a graphical user interface.

## 3. Functions

### 3.1. Interview survey

First of all, we conducted an interview survey to identify challenges that novice users face in the experimental process of photoemission spectroscopy. Users who had experience with photoemission spectroscopy experiments participated in this survey, including experts and students (non-expert) in the materials science research field. We asked two experts and five students, where the experts have used photoemission spectroscopy with synchrotron radiation for more than ten years. In the interviews we asked the interviewees common questions such as about their experience of synchrotron radiation experiments, the series of events in the experiments, what is the bottleneck in photoemission spectroscopy experiments and how would they solve/handle it. The survey results revealed two major problems when performing photoemission spectroscopy experiments: peak identification and SNR determination.

The first problem to mention is peak identification. In photoemission spectroscopy, spectral peaks are important information because the positions of peaks indicate what kinds of elements are contained in the samples. Peaks mainly derive from photoelectrons of the sample – electrons emitted from the inner shells of the elements due to the photoelectric effect. Other possible sources of peaks are signals from extrinsic polar molecules absorbed on the surface and Auger electrons due to core-hole excitations. Participants of the interview survey pointed out that it is difficult to distinguish between photoemission spectrum peaks that come from the constituent elements of the samples and other peaks. Also, in some cases, errors in measurement were only noticed later, or errors were not noticed in the first place. This problem is particularly serious in measurements using synchrotron radiation because if measurements are missed then they may not be able to be taken again for maybe half a year due to the limited beam time access for photoemission spectroscopy experiments at synchrotron sources.

The other problem is the SNR. The SNR of spectra is increased by repeating measurements under the same conditions and aggregating them. However, when to stop the iteration is determined visually and empirically from the resulting spectra. In particular, the use of synchrotron radiation as a light source makes it more difficult for novice users to carry out photoemission spectroscopy experiments because if they obtain spectra that do not reach the necessary SNR levels they would have to conduct the measurement again maybe half a year later due to limited beam time access as mentioned above.

### 3.2. Function design

Given the two major problems found by the interview survey, we decided to implement the following three functions in *synapse*:

(1) Function to formulate and visualize the SNR.

(2) Function to display the binding energy of the constituent elements of the sample on the spectrum.

(3) Function to manage meta information and data in the form of log notes.

First, we propose a method for calculating the SNR. Currently the SNR is judged visually and empirically from spectra, but it would be better if it could be evaluated quantitatively. Also, the SNR should not only be calculated but also visualized so that users can quickly grasp it.

In addition, we show on the spectrum the binding energies of the elements that might possibly be contained in the sample. Currently, users have to reference data tables of binding energies in order to help identify the origin of the peaks. Therefore, they have to search data from a large database (often in the form of physical books), making it difficult for unfamiliar users to identify the peaks – having the binding energy showing on the spectra would make it easier to work out their origin.

We also organize the measurement information of the experiments following the form of existing log notes, in which experimenters record the values of the parameters used and the experimental results, such as the sample position, intensity of light and presets used in the measurement. By adopting this log note format, it is expected that users will be able to use the application according to their existing user experience.

## 4. Implementation

### 4.1. Architecture

In this section, we discuss how to build the entire application, not each function. This section is divided into the data input/output part, the logic part and the interface part, and is explained in detail.

In many cases, there is no application programming interface (API) or software development kit provided for photoemission spectroscopy instruments. Even if this is available, it is often limited to *LabVIEW* [graphical programming environments developed by National Instruments Corporation (https://www.ni.com/ja-jp/shop/labview.html)] (Kalkman, 1995 ▸). Therefore, we implemented a system such that data files are loaded and processed after measurement. This is the same as when processing is carried out by analysis software such as *Igor* [software for analyzing spectra provided by Wavemetrics (https://www.hulinks.co.jp/software/da_visual/igor/section02)], so the existing user experience is not compromised.

**Figure 1:**
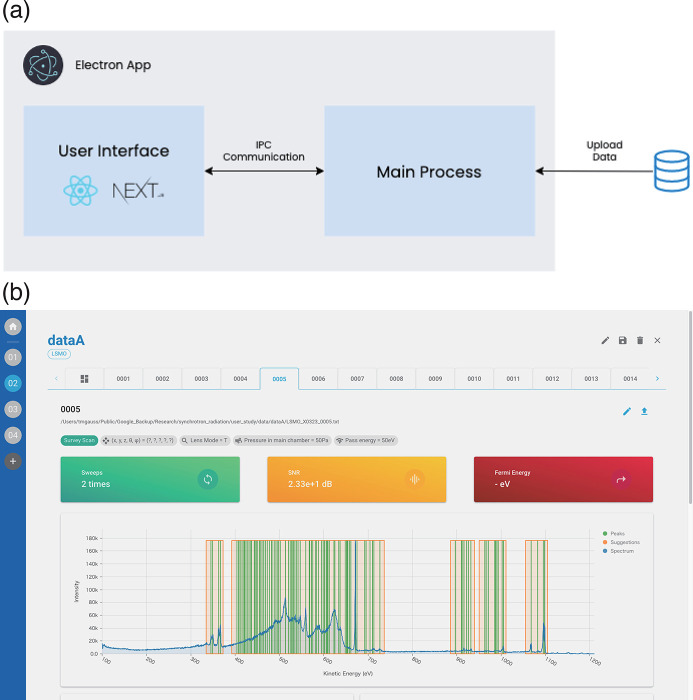
The graphical user interface of *synapse*. (*a*) Architecture. (*b*) User interface.

The core logic of *synapse* was basically implemented in TypeScript, which is a strongly typed programming language that builds on JavaScript. We use various packages provided by the NPM community to save time and effort in implementation. For example, *synapse* is built on *Electron*, a framework enabling developers to build cross-platform desktop apps, Next.js (https://nextjs.org/), a React (https://ja.reactjs.org/) framework facilitating static site generation and server-side rendering, and MUI [a front-end package for building user interfaces based on UI components maintained by Meta (formerly Facebook)], a React component library with material design (the design scheme developed by Google). The architecture of *synapse* is shown in Fig. 1 ▸(*a*) (see also Appendix *A* [App appa]). In the main process, *synapse* communicates with the file API, which enables users to read or write data files. Via inter-process communication (IPC), the main process communicates with the renderer process, in which the application shows the user interface.

The application interface was also basically implemented in TypeScript. We use Next.js to improve the performance of the application and MUI to save time developing React components from scratch. The interface is shown in Fig. 1 ▸(*b*). The following is a brief description of how to use the system. First, create a project and enter the names and constituent elements of the samples to be used. Then, add measurement data along with metadata about the equipment, sample location, light source, *etc*. After that, the dashboard is displayed, in the center of which the spectrum is displayed with the binding energies of the constituent elements. Above that, accumulations, SNR and Fermi levels are visualized.

### 4.2. Formula of the SNR

Currently, there is no prevalent method for calculating the SNR in photoemission spectroscopy. Therefore, we first list the requirements that the SNR must meet: (i) dimensionless; (ii) ability to be compared across spectra; (iii) monotonically increasing according to the number of iterations. To achieve these requirements, we decided to compute the SNR using the Savitzky–Golay filter (Savitzky & Golay, 1964 ▸), a smoothing filter that can reduce noise.

Generally, a smoothing filter modifies each data point using its adjacent points. One of the simplest smoothing filters is the moving average filter, which converts a certain data point into an average of its adjacent points. Let *x*_*i*_ be the *i*th data point and 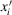 the point converted from *x*_*i*_ by a moving average filter. Then, 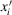 can be described as follows, 

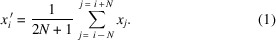

Here, 2*N* + 1 is the window length, which means that the point, the *N* points to the left of it, and the *N* points to the right of it are used. The Savitzky–Golay filter is very similar to the moving average filter. While a moving average filter replaces a data point with the mean of its adjacent points, the Savitzky–Golay filter replaces a data point with a central point of the polynomial curve that fits its adjacent points by the method of linear least squares. Its parameters are window length and polynomial order.

Let *I*_*i*_ (*i* = 0, 1, 2,…, *N* − 1) be the intensity of the *i*th data point. Let *I*_*i*,net_ (*i* = 0, 1, 2,…, *N* − 1) be the discrete data of *I*_*i*_ to which the Savitzky–Golay filter is applied. Based on the results of Furukawa’s experiment (Furukawa *et al.*, 2016 ▸), we let the window length be 11, while, based on our preliminary experiment, we temporally let the polynomial order be 4. Also, we define the noise as follows, 

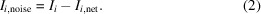

Using these, we define the SNR as follows,

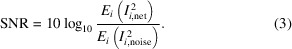

Note that 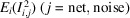 represents the average of 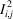 and has units of dB, which is of course a dimensionless quantity.

### 4.3. Algorithm of peak identification

As a preliminary experiment, we tried two implementable methods for peak identification: the threshold curve of the second derivative method (2nd DER method) and the directly calculating peak and background relations at a candidate peak method (PB method) (Furukawa *et al.*, 2016 ▸). As a consequence, we found that it is difficult to distinguish between broad peaks and large noise in the 2nd DER method, and the PB method hardly identifies peaks with narrow widths and low intensities from noise. Considering these results, we have tried to develop the PB method to identify the peaks based on their intensities and widths.

The algorithm of the peak identification in *synapse* runs as follows. Firstly, the maximum values of the peak candidates are estimated after subtracting the background, where the background is subtracted using the Shirley method. Secondly, the peak candidates with intensities higher than twice the value of the standard deviation of the noise are regarded as the peaks. Finally, after the intensity cutoff, the peak candidates with widths narrower than 0.5 eV are considered as the noise. Here, the peak width is half the value of the full width. We have employed the *X-ray Data Booklet* (Thompson *et al.*, 2009 ▸) as reference for the binding energy data.

The photon energy is a parameter used to identify the binding energies of the photoemission spectra. To correct the binding energy, the value of the Fermi level estimated from the Fermi edge of gold that electrically contacts to the sample is used. The Fermi edge is measured when the photon energy is changed.

## 5. Evaluation

To evaluate *synapse*, we conducted two types of evaluation experiments – a quantitative evaluation and a qualitative evaluation. First, we quantitatively evaluated the formula of the SNR, while qualitatively investigating the peak identification and the user experience of *synapse*. This section describes these evaluations.

### 5.1. Quantitative evaluation of the formulation of the SNR

**Figure 2:**
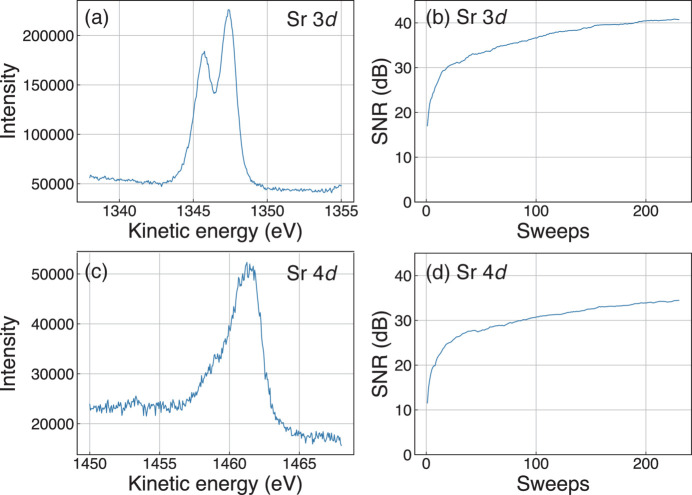
Evaluation of the SNR. (*a*) Data on the spectrum of the 3*d* orbital of Sr. The number of accumulations is 230. (*b*) SNR of 3*d* orbitals of Sr. The horizontal axis is the number of accumulations and the vertical axis is the SNR. (*c*) Data on the spectrum of the 4*d* orbital of Sr. The number of accumulations is 230. (*d*) SNR of 4*d* orbitals of Sr. The horizontal axis is the number of accumulations and the vertical axis is the SNR.

We applied the methods described in Section 4.2 to actual data and plotted intensity and SNR according to kinetic energy and sweeps, respectively. We used a window width of 11 and a polynomial degree of 4 for the parameters of the filter, referring to the work of Furukawa *et al.* (2016 ▸). The results are shown in Fig. 2 ▸. Judging visually, it is clear that the SNR of the Sr 3*d* spectrum is higher than that of the Sr 4*d* spectrum, as shown in Figs. 2 ▸(*a*) and 2(*c*). This is consistent with the results comparing the SNRs of the rightmost point on the right-hand side of Fig. 2 ▸(*b*) and that of Fig. 2 ▸(*d*). Furthermore, it is confirmed that the SNR increases with increasing number of accumulations, as shown in Figs. 2 ▸(*b*) and 2 ▸(*d*).

### 5.2. Qualitative evaluation of peak identification

**Figure 3:**
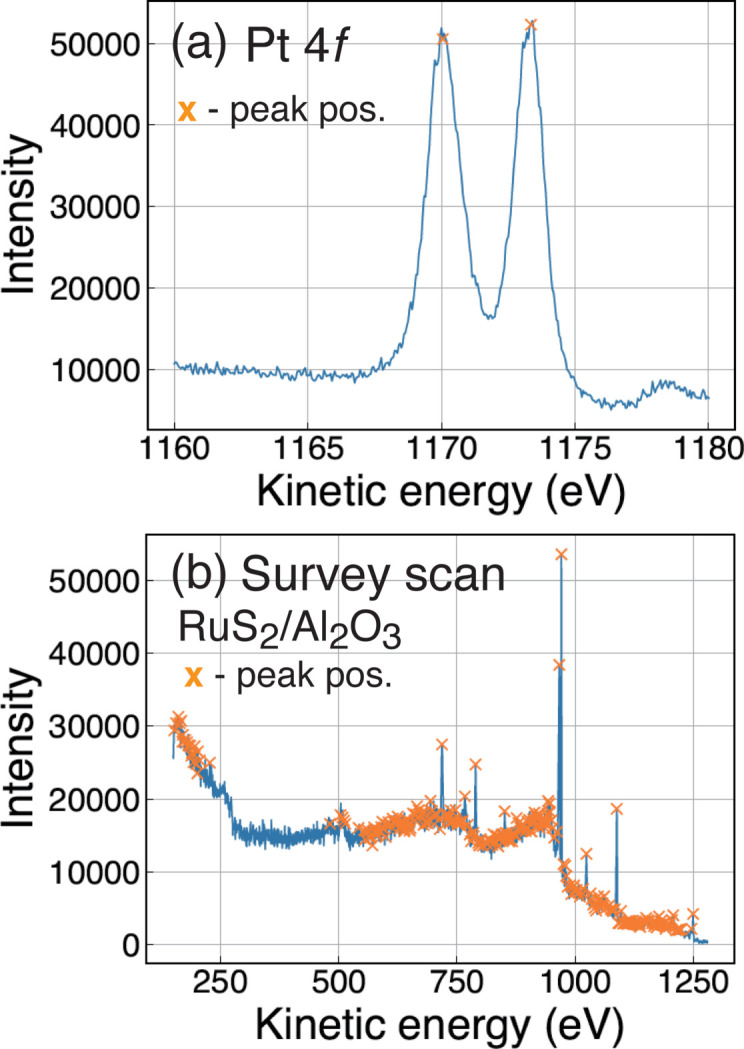
Evaluation of the peak identification. (*a*) Pt 4*f* spectrum. (*b*) Survey scan of Ru_2_/Al_2_O_3_. The cross marks are the peaks identified by *synapse*.

Figure 3 ▸ shows results of peak identifications for a core-level spectrum and wide scan. The positions of the main peaks are well identified by this algorithm, while not only the core-level peaks but also the non-core-level peaks are evaluated as peaks, as shown in Figs. 3 ▸(*a*) and 3 ▸(*b*).

Additionally, to evaluate the algorithm of the peak identification, we have attempted to identify peaks for 22 core-level spectra and six wide scans (survey scans). For the core-level spectra, the peak identification for the main peaks works well, while it is difficult to identify the satellite and overlapped peak structures. As a result, the total accuracy rate is about 40%. For the survey scans, it seems to be difficult to distinguish between the peaks and noise. This may come from the low SNR and peak structures with relatively low intensities. Then, the algorithm of the peak identification will be improved to identify peaks with high accuracy rate and distinguish additional structures. In the present version of *synapse*, the observed elements can be identified at least.

### 5.3. Qualitative evaluation of user experience

We conducted a user study in which one expert and one student were asked to use the application and then interviewed about their experience. In the experiments, the expert made actual measurements and analyzed the data at a synchrotron radiation facility; on the other hand, the experiment of the student was a simulated analysis using pre-measured data. Afterwards, we conducted interviews in which the questions mainly focused on the following point: ‘Is it easy to stop the repetition of the measurements (Function 1)?’. The format of the interview was a semi-structured interview based on the following questions prepared in advance, with further questions asked according to the participants’ answers:

(i) Did you require less effort to perform the analysis using this application?

(ii) How has your performance changed using this application?

(iii) Compared with the existing interface, is the new interface easier to use?

(iv) Would you recommend this application to someone you know?

(v) In addition to the previous answers, what aspects of the system have you found easier to use?

(vi) In addition to the previous answers, what improvements should be made?

(vii) Did you find the measurement after the survey scan to be clear? Please answer with the reason.

(viii) Has the SNR been clarified? Please answer along with your reasons.

As a result, the opinions were very positive about the ability to quantitatively calculate and visualize SNRs (Function 1). The expert said that this function is the best part of all the functions of *synapse* because people without experience do not know how much experimental data they need to accumulate. The student added that if the SNR is expressed in numbers it gives an indicator of how many iterations users should take.

Other than Function 1, opinions were obtained about the function to display the binding energy (Function 2) and the function to organize information in the form of log notes (Function 3). About Function 2, they expressed that the labor was reduced by annexing the binding energies of the constituent elements of the sample to the spectra. The student commented that the energy positions are quite easy to see because they appear together on the screen, meaning that users do not have to remember or check binding energies each time. Also, the participants gave the opinion that compiling information in the form of log notes saves time and reduces the risk of errors. The expert said that, if the software shows us what information is actually required, it is easier for people who have no experience to understand what information is needed. The student also added that it would be great to be able to go with the flow and not make mistakes just by taking a log note.

Other opinions about the interface were as follows:

(i) The filtering algorithm seems to be a ‘black box’. It is difficult to judge whether the smoothing is optimized or whether there is space for improvement.

(ii) It is better to visualize a filtering method and change the degree of the smoothing manually.

(iii) It is better to treat not only 1D data but also 2D data taken by using angle-resolved photoemission spectroscopy (ARPES). Please extend these functions for ARPES.

## 6. Discussions

The previous section describes the experiments in which we evaluate *synapse* in terms of quantity and quality. In this section the results of evaluation experiments and limitations and future work are discussed.

### 6.1. Discussion of SNR determination

As shown in the interview survey (Section 3.1), it is not easy for novice users to determine the necessary and sufficient SNR. Therefore, we formulated the SNR using equation (3) and visualized it as a guideline for determining the necessary and sufficient SNR.

The results of the experiments described in Section 5.1 suggest that the definition in equation (3) is valid and satisfies all three requirements mentioned in Section 4.2: dimensionless, ability to be compared across spectra, and monotonically increasing according to the number of iterations. Given equation (3), it is clear that the SNR is dimensionless. Also, as shown in Figs. 2 ▸(*a*) and 2 ▸(*c*), the SNR of Sr 3*d* spectra is obviously larger than that of Sr 4*d* spectra, and this intuition is consistent with the calculation. Finally, given the spectra shown in Figs. 2 ▸(*b*) and 2 ▸(*d*), calculated SNRs are almost monotonically increasing. Thus, we could conclude that the suggested formula of the SNR can be a great indicator for users of when to stop the iterations.

Furthermore, the results of the user study reveal that calculating and visualizing SNRs helps novice users of photoemission spectroscopy. This means that the two findings do not only prove how valid the definition of SNR is but also how effective defining the SNR itself is. In the past, novice users have had to ask experienced users for help when they determine the SNR, but, from now on, veteran users can leave the measurement, for example, by saying, ‘Keep it running until it reaches 40 dB’. Certainly, further discussion and consensus-building are necessary regarding the validity of equation (3) and the type and parameters of the filter to be used, but SNR visualization is considered to be a sufficient solution to the issue of photoemission spectroscopy.

In the present version of *synapse*, the value of the SNR is estimated based on the intensity. It seems to be possible to estimate the SNR based on the different peak-to-background intensity by subtracting the background before the estimation. In this case, the value of the SNR possibly becomes an absolute basis for judging the noise level. This is a future work for *synapse*.

### 6.2. Limitations and future work

In this section we provide four limitations in this study: filters, data sets, data loading and application to other measurements.

The selection of filters can be a limitation. A Savitzky–Golay filter was used in this study. However, there are other noise-reducing filters other than the Savitzky–Golay filter, such as moving average filters and simple low-pass filters. There seems to be room for discussion regarding the type of filter and parameters used to calculate the SNR; if the SNR is not standardized and there are multiple definitions, there is a risk of further confusion for non-skilled users.

Also, the data sets we used in this study are limited in number and do not cover all cases. In photoemission spectroscopy, there is no common database that stores experimental data, which limits the data our team has and prevents us from freely debugging and validating the algorithms. Therefore, for future algorithm development it will be necessary to construct a common data set for photoemission spectroscopy.

The third limitation is how to load the data. The current version of *synapse* requires data files to be read one by one. As it is, it is not easy to determine SNRs in real time. Ideally, a file should be monitored for changes and automatically updated. A file monitoring function is relatively easy to implement and will be incorporated into the application in the future.

Lastly, in this study, we focus on ordinary photoemission spectroscopy. As an option, we would like to expand *synapse* to ARPES, which is becoming more popular in materials science. Indeed, the participants of the user study recommend the expansion of the target.

## 7. Conclusion

In this study, we proposed a measurement and analysis support system for users who are not familiar with photoemission spectroscopy. First, we conducted interviews and found two major issues in the experimental process of photoemission spectroscopy: peak identification and SNR determination. In order to solve these problems, we have developed *synapse* with the following three functions: function to quantitatively calculate and visualize SNRs, function to record the binding energies of the constituent elements of a sample, and function to manage information in the form of log notes. Finally, the application was evaluated quantitatively and qualitatively. It was found that the SNR could be determined easily enough, although there is room for improvement to assist in peak identification.

In the future, we aim to solve the issues described in Section 6.2 and build an application that is easier to use for novice users of photoemission spectroscopy.

## Appendix A Architecture

*synapse* is built using *Electron*, a JavaScript package to build cross-platform desktop apps. Using *Electron*, we can easily build native desktop apps instead of web-based apps activating on browsers, which avoids users having to start apps by command-line interfaces. Here, *Electron* and its multi-process model are explained.

### A1. An overview of *Electron*

*Electron* (https://www.electronjs.org/ja/docs/latest) can be characterized by three points: compatibility, web-based stacks and use cases. *Electron* enables developers to readily build native apps compatible with the various operating systems: Mac, Windows and Linux. Also, *Electron* can be used with HTML, CSS and JavaScript, which are the stacks used in the development of web-based apps (Type-Script, React and any other packages essential to modern front-end development can be used). Thus, *Electron* is trusted by VSCode, WhatsApp, Slack, Figma, Twitch and so on.

### A2. A multi-process model of *Electron*

*Electron* applications have a multi-process model, where developers can use the main process and renderer processes. In short, the main process orchestrates multiple renderer processes, which are responsible for the user interface. The main process is unique to the *Electron* app and plays a role in activating the *Electron* app, managing renderer processes (app windows) and corresponding with operating systems. On the other hand, the renderer processes play a role in the rendering interface, behaving according to web standards. Thus, we can easily and quickly migrate from web-based apps to native apps. The main process communicates with the renderer processes via IPC. Using IPC, the renderer processes obtain access to operating systems such as file systems.

The renderer processes act like browser tabs, so developers can have the benefit of numerous JavaScript packages including React, Next.js, TypeScript and MUI. In that respect, web-based app development is not different from native app development. If we want to move from web-based apps to native apps, all we have to do is to transplant client parts to renderer processes and server parts to the main process.
